# Hyaluronic Acid Injections Are Associated with Delay of Total Knee Replacement Surgery in Patients with Knee Osteoarthritis: Evidence from a Large U.S. Health Claims Database

**DOI:** 10.1371/journal.pone.0145776

**Published:** 2015-12-22

**Authors:** Roy Altman, Sooyeol Lim, R. Grant Steen, Vinod Dasa

**Affiliations:** 1 Department of Rheumatology, University of California Los Angeles, Los Angeles, California, United States of America; 2 North American Business Unit, Seikagaku Corporation, Tokyo, Japan; 3 Department of Medical Affairs, Bioventus LLC, Durham, NC, United States of America; 4 Department of Orthopaedics, Louisiana State University Health Sciences Center, New Orleans, Louisiana, United States of America; University of Texas Health Science Center at Houston, UNITED STATES

## Abstract

**Background:**

The growing prevalence of osteoarthritis (OA) and the medical costs associated with total knee replacement (TKR) surgery for end-stage OA motivate a search for agents that can delay OA progression. We test a hypothesis that hyaluronic acid (HA) injection is associated with delay of TKR in a dose-dependent manner.

**Methods and Findings:**

We retrospectively evaluated records in an administrative claims database of ~79 million patients, to identify all patients with knee OA who received TKR during a 6-year period. Only patients with continuous plan enrollment from diagnosis until TKR were included, so that complete medical records were available. OA diagnosis was the index event and we evaluated time-to-TKR as a function of the number of HA injections. The database included 182,022 patients with knee OA who had TKR; 50,349 (27.7%) of these patients were classified as HA Users, receiving ≥1 courses of HA prior to TKR, while 131,673 patients (72.3%) were HA Non-users prior to TKR, receiving no HA. Cox proportional hazards modelling shows that TKR risk decreases as a function of the number of HA injection courses, if patient age, gender, and disease comorbidity are used as background covariates. Multiple HA injections are therefore associated with delay of TKR (all, P < 0.0001). Half of HA Non-users had a TKR by 114 days post-diagnosis of knee OA, whereas half of HA Users had a TKR by 484 days post-diagnosis (χ^2^ = 19,769; p < 0.0001). Patients who received no HA had a mean time-to-TKR of 0.7 years; with one course of HA, the mean time to TKR was 1.4 years (χ^2^ = 13,725; p < 0.0001); patients who received ≥5 courses delayed TKR by 3.6 years (χ^2^ = 19,935; p < 0.0001).

**Conclusions:**

HA injection in patients with knee OA is associated with a dose-dependent increase in time-to-TKR.

## Introduction

The age-standardized prevalence of knee osteoarthritis (OA) is 3.8%, making OA one of the leading causes of global disability [1]. Intra-articular (IA) injection of hyaluronic acid (HA) has been recommended to alleviate pain and improve joint function in patients with knee OA [2]. Chondroprotective and analgesic properties inherent to HA [3] suggest that HA can delay total knee replacement (TKR) surgery [4], a treatment popular enough that it has become a key driver of health care costs [5]. It is estimated that 54% of knee OA patients will receive TKR over their lifetime under current guidelines; the current trend of expanding indications for TKR suggests that there may be a 29% increase in lifetime direct medical costs attributable to TKR among knee OA patients [6].

Recent OA treatment guidelines from Europe recommend that HA be used for symptom relief prior to TKR [7], whereas the American Academy of Orthopedic Surgeons (AAOS) recently recommended against using HA [8]. Considering a standardized patient with moderate knee pain and functional limitations, orthopedic surgeons and rheumatologists differ significantly in their recommendations for TKR [9] underscoring the need for further evidence to formulate rational OA treatment algorithms. Published clinical studies generally evaluate the efficacy of HA in reducing pain, with observation periods that rarely exceed 6 months. Yet the proposed mode of action of HA warrants investigation of its potential role in delaying the progression of disease over longer periods of time [10].

There are currently no completed, ongoing, or recruiting randomized clinical trials (RCTs) designed to determine whether HA can delay or prevent TKR [11]. The Agency for Healthcare Research and Quality (AHRQ) recently reviewed the published literature on HA injections with the aim of identifying any role in delaying TKR surgery. Given the absence of relevant RCTs, the AHRQ advocated analyzing data from a real-world administrative claims database to test whether HA use can delay TKR [11].

To evaluate the impact of HA use on TKR, we evaluated every patient with knee OA who received TKR in a database of approximately 79 million patients. Our hypothesis was that HA injection is associated with delay of TKR in a dose-dependent manner, such that more courses of HA will delay TKR for a longer period of time. The primary outcome was a comparison of time-to-TKR in patients who received HA, relative to patients who did not receive HA. The secondary outcome was to examine the relationship between number of HA injections and time-to-TKR.

## Methods

This retrospective claims analysis used the IMS Health PharMetrics Plus database of ~79 million patients. Because all patient data were anonymized before analysis, no Institutional Review Board (IRB) approval was required. Adjudicated health claims were aggregated from many sources; patients in each 3-digit zip code and in every Metropolitan Statistical Area of the United States are included, with coverage of data from 90% of American hospitals, 80% of American doctors, and 85% of Fortune 100 companies [12].

We identified all knee OA patients who received TKR within a 6-year selection window (2007–2013). We evaluated only those knee OA patients who had continuous enrollment from OA diagnosis until TKR. The initial OA diagnosis served as the index date and we evaluated time-to-TKR, in days from diagnosis, as a function of the number of courses of HA injection received, ranging from 0 to ≥5. To evaluate differences in demographic factors between HA Users and Non-users, we used χ^2^ to evaluate categorical variables and Wilcoxon rank sum for continuous variables. We also did a Cox proportional hazards analysis, to evaluate the impact of number of courses of HA injection, age (as a continuous variable), gender, and comorbidity (as a continuous variable) on the risk of TKR. Time-to-event analyses used a Kaplan-Meier survival approach. Median TKR-free survival time was defined as the time at which 50% of patients in a cohort received TKR. Log-rank tests were used to compare TKR-free survival times for the different HA cohorts. All statistical analyses were performed using SAS 9.2 (SAS Institute; Cary, NC).

## Results

**Fig 1:**
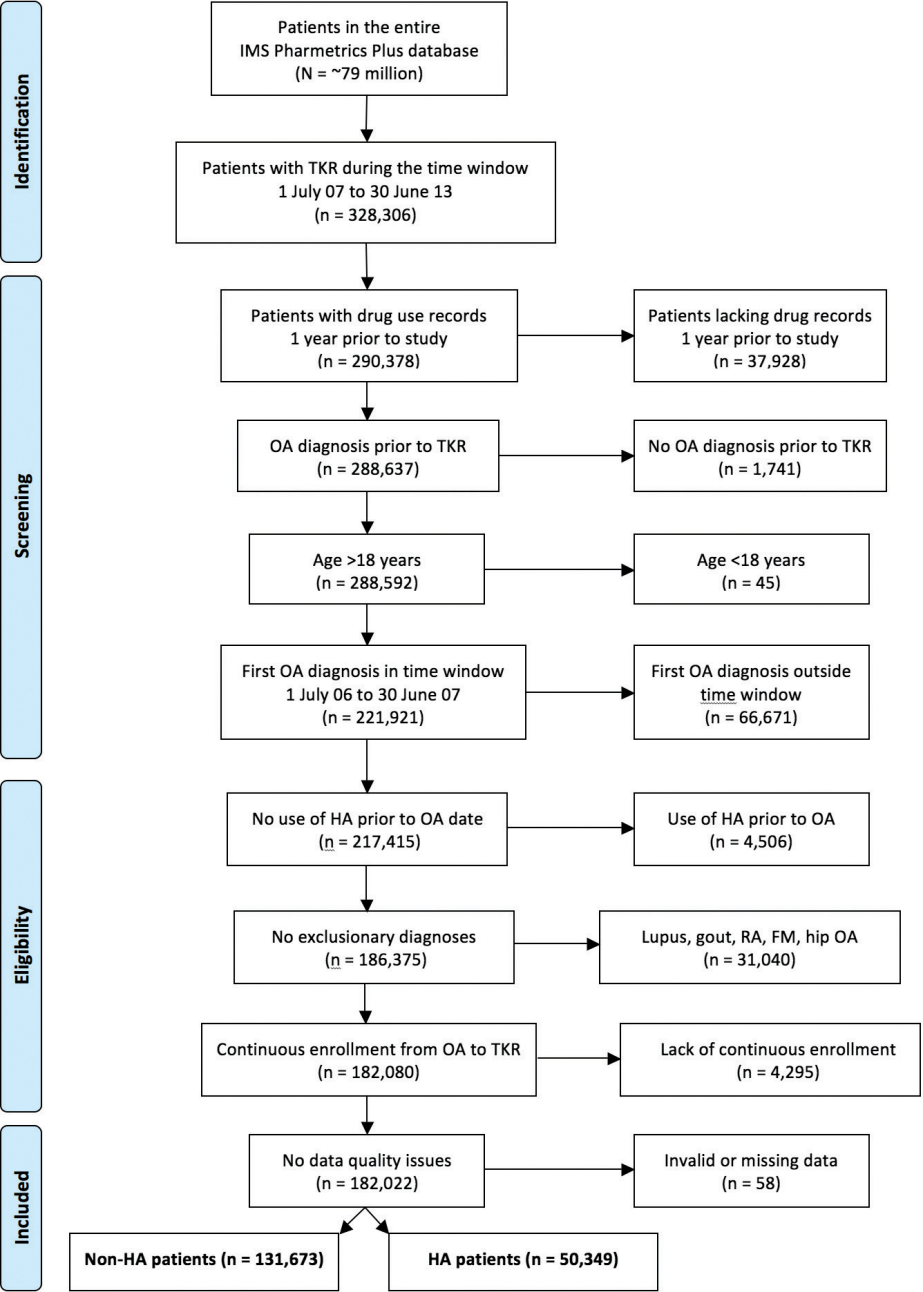
PRISMA-style diagram. This Fig summarizes how patients were accrued to the study.

The database of ~79 million patients yielded 328,306 patients who had TKR between July, 2007 and June, 2013 (Fig 1). After excluding patients lacking an OA diagnosis, lacking continuous enrollment, lacking drug records, or having various exclusionary diagnoses (lupus erythematosus, gout, rheumatoid arthritis, fibromyalgia, hip OA), the database included 182,022 patients with both knee OA and TKR who met all inclusion criteria. Roughly 55% of possible TKR patients (= 182,022/328,306) are included in the present analysis (Fig 1).

A total of 50,349 (27.7%) patients were classified as HA Users, receiving 1 or more courses of HA, while 131,673 patients (72.3%) were classified as HA Non-users (Table 1). Age, gender, and Charlson Comorbidity Index (CCI) scores differed significantly between patients who did and did not use HA (Table 1), though sample sizes in this database are so large that differences that are not clinically important can still be statistically significant. Patients who received a TKR without first using HA were on average 1.8 years older than patients who used HA prior to TKR, with HA Non-users involving fewer patients in the age 45 to 54 stratum and more patients in the age >65 stratum. HA Non-users were also more likely to be men, less likely to be self-insured, and more likely to consult an orthopedic surgeon (Table 1).

**Table 1:** Demographic descriptors of HA Users and HA Non-users. The P value shown is for a χ^2^ evaluation of all demographic descriptors in the category. The heading “GP/FP/IM” includes physicians in General Practice, Family Practice, and Internal Medicine.

	HA Patients		Non-HA Group		
Descriptors	N = 50,349		N = 131,673		P-value
**Age (n,%):**					
18–44 years	1,713	3.4%	2,966	2.3%	< .0001
45–54	12,193	24.2%	23,563	17.9%	
55–64	24,034	47.7%	63,540	48.3%	
65+	12,409	24.6%	41,604	31.6%	
Mean	59.7		61.5		< .0001
SD	8.9		8.9		
**Gender (n,%):**					
Male	19,386	38.5%	57,628	43.8%	< .0001
**Payer Type (n,%):**					
Commercial Plan	29,770	59.1%	79,036	60.0%	< .0001
Medicaid	241	0.5%	884	0.7%	
Medicare Risk	1,759	3.5%	6,348	4.8%	
Medicare Cost	934	1.9%	2,973	2.3%	
Self-Insured	17,328	34.4%	41,708	31.7%	
Unknown	317	0.6%	724	0.5%	
**Physician Specialty (n,%):**					
Orthopedic Surgery	22,051	43.8%	59,547	45.2%	< .0001
GP/FP/IM	5,212	10.4%	13,436	10.2%	
Orthopedics	3,486	6.9%	9,376	7.1%	
Rehab. Medicine	950	1.9%	1,416	1.1%	
Rheumatology	722	1.4%	838	0.6%	
Other	17,928	35.6%	47,060	35.7%	
**Charlson Comorbidity Index (n,%):**					
0	32,992	65.5%	84,797	64.4%	< .0001
1	9,036	17.9%	23,943	18.2%	
2	5,134	10.2%	13,789	10.5%	
3	1,837	3.6%	5,189	3.9%	
4+	1,350	2.7%	3,955	3.0%	
**Mean CCI**	0.6		0.7		< .0001
SD	1.1		1.1		
Median	0		0		

A Cox proportional hazards model shows that the risk of having TKR decreases as a function of the number of courses of HA injection, if patient age (as a continuous variable), patient gender, and patient comorbidity (as a continuous variable) are added as background covariates (Table 2). Our analysis shows that "HA 1 course vs non HA cohort" significantly delays TKR (P < 0.0001) after controlling for age, gender, and CCI. The hazard ratios for number of HA courses are all statistically significant and less than one, suggesting that HA injection is protective from the risk of TKR (all, P < 0.0001). The hazard ratios for age and gender are also statistically significant, but do not influence risk of TKR as much as does HA injection. CCI, treated as a continuous variable, does not significantly increase the risk of TKR (Table 2). These findings suggest that, while there are clinical differences between HA users and HA non-users in background covariates, an important difference is in HA use itself.

**Table 2:** Risk of TKR as a function of number of courses of HA injection, age, gender, and comorbidity. Coeff. is the coefficient of a Cox proportional hazards model; a negative value indicates that the variable is protective from the risk of TKR. Comorbidity is expressed as the Charlson Comorbidity Index (CCI). The P value shown is for a χ^2^ evaluation of each independent variable.

		Standard	Hazard	95% CI		
Independent Variables	Coeff.	Error	Ratio	Lower	Upper	**P-value**
HA 1 course v. non-HA cohort	-0.571	0.006	0.565	0.559	0.572	< .0001
HA 2 courses v. non-HA cohort	-0.901	0.011	0.406	0.397	0.415	< .0001
HA 3 courses v. non-HA cohort	-1.132	0.019	0.322	0.310	0.335	< .0001
HA 4 courses v. non-HA cohort	-1.265	0.031	0.282	0.266	0.300	< .0001
HA ≥5 courses v. non-HA cohort	-1.496	0.036	0.224	0.209	0.241	< .0001
Age (continuous variable)	0.010	0.000	1.010	1.009	1.010	< .0001
Gender: male vs. female	0.042	0.005	1.043	1.033	1.053	< .0001
CCI (continuous variable)	-0.002	0.002	0.998	0.994	1.003	0.4328

Among HA Users, 73.2% received 1 course of HA treatment, 17.7% received 2 courses, 5.5% received 3 courses, 2.1% received 4 courses, and 1.5% received ≥5 courses (Table 3). Median time-to-TKR increased from 114 days for patients with no use of HA to 484 days for patients who received any HA injection (Table 3). Time from OA diagnosis to when 50% of patients received TKR was significantly (p<0.0001) and substantially longer for HA Users, compared to HA Non-users. Half of HA Non-users had a TKR at 114 days post-diagnosis, whereas half of HA Users had a TKR at 484 days post-diagnosis (χ^2^ = 19,769; p < 0.0001), more than 4 times longer than the median time-to-TKR of HA Non-users (Table 3).

**Table 3:** Timing of total knee replacement (TKR) for all patients. Patients in the HA cohort got a TKR on average 601.8 days after OA diagnosis, which is significantly longer than the mean time-to-TKR of 270.3 days in patients who did not receive HA (χ^2^ = 19,769.1; p < 0.0001).

		Sample	Median	Mean	SD		
		N =	days	days	days	χ^2^ =	P =
**Non-HA cohort**		131,673	114	270.3	355.5	-	-
**HA cohort**		50,349	484	601.8	433.2	19,769.1	<0.0001
	1 HA course	36,861	386	513.7	400.1	13,724.6	<0.0001
	2 HA courses	8,893	648	741.7	408.6	18,138.7	<0.0001
	3 HA courses	2,783	875	945.6	390.5	19,363.5	<0.0001
	4 HA courses	1,052	1,054	1,085.1	356.1	19,664.4	<0.0001
	≥5 HA courses	760	1,312	1,306.0	355.7	19,934.6	<0.0001

Disease comorbidities and medication use at or before OA diagnosis were similar between HA Users and HA Non-users (Table 4). Several illnesses were somewhat more prevalent among HA Users, including hyperlipidemia, major depression, bipolar disorder, liver disease, and substance abuse disorder, while schizophrenia and anemia were equally prevalent between HA Users and HA Non-users. HA Non-users were somewhat more likely to have hypertension, diabetes, vascular disease, renal disease, and chronic obstructive pulmonary disease. HA Users used somewhat more of all medications at or before OA diagnosis, including corticosteroids, non-steroidal anti-inflammatory drugs (NSAIDs), opioids, and proton-pump inhibitors.

**Table 4:** Summary of comorbidities and medications among HA Users and HA Non-users.

	HA Users		HA Non-Users	
	N = 50,349		N = 131,673	
**Comorbidities of Interest (n,%):**				
Hypertension	22,991	45.7%	61,494	46.7%
Hyperlipidemia	21,236	42.2%	53,197	40.4%
Diabetes	8,251	16.4%	23,443	17.8%
Schizophrenia	54	0.1%	105	0.1%
Major depression	1,534	3.0%	3,171	2.4%
Bipolar disorder	473	0.9%	984	0.7%
Vascular disease	12,479	24.8%	33,568	25.5%
Liver disease	1,238	2.5%	2,764	2.1%
Renal disease	2,563	5.1%	7,092	5.4%
Substance abuse disorder	1,569	3.1%	3,921	3.0%
Chronic obstructive pulmonary disease	1,687	3.4%	4,707	3.6%
Anemia	3,254	6.5%	8,572	6.5%
**Medications of Interest (n,%):**				
Corticosteroids	14,296	28.4%	27,407	20.8%
NSAIDs	14,112	28.0%	29,953	22.7%
COX-2 inhibitors	3,149	6.3%	6,977	5.3%
Analgesics non-narcotic	619	1.2%	1,229	0.9%
Opioids	17,763	35.3%	40,980	31.1%
Non-NSAID analgesics	149	0.3%	290	0.2%
H^2^ blockers	1,056	2.1%	2,521	1.9%
Proton pump inhibitors	9,271	18.4%	20,876	15.9%

**Fig 2:**
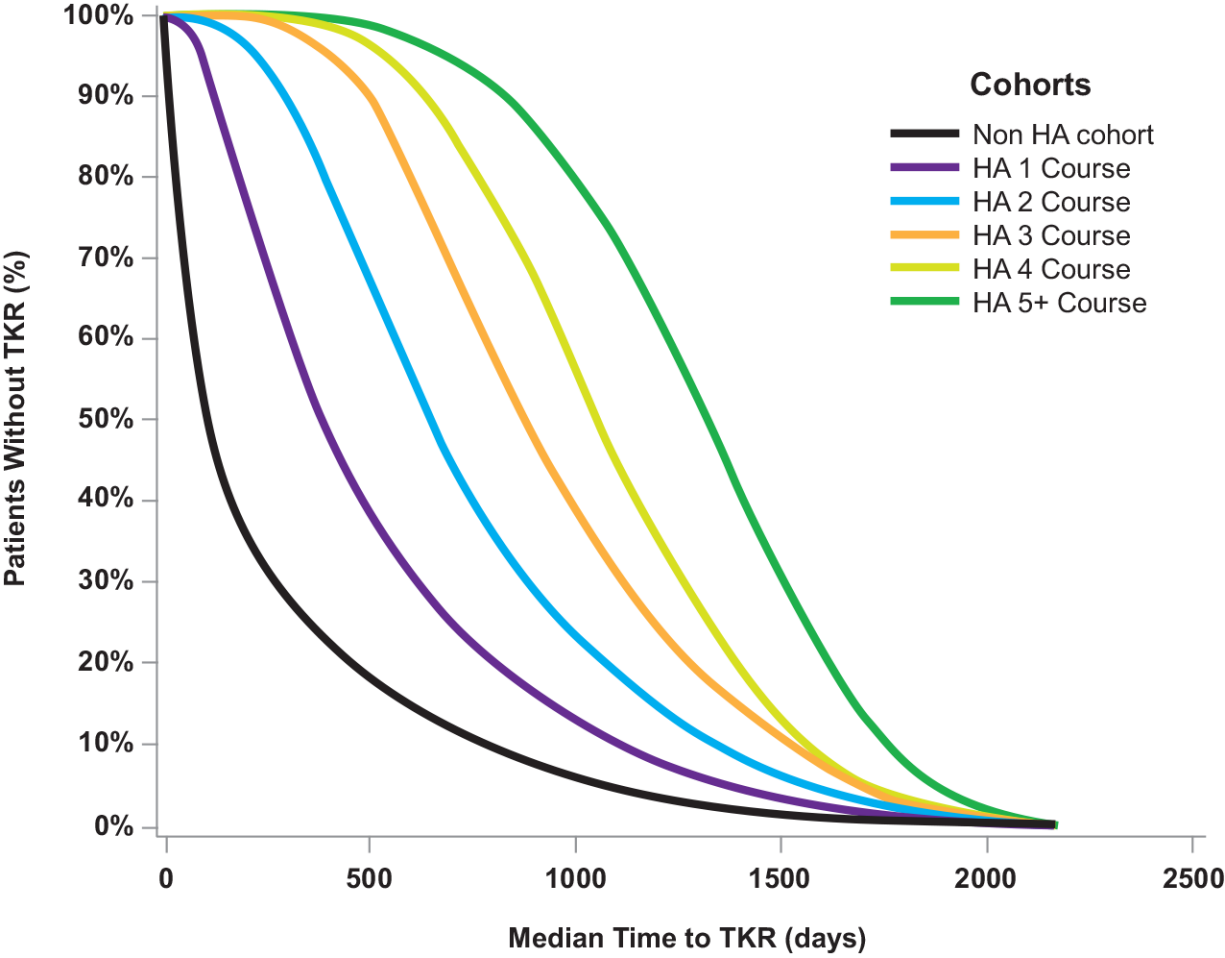
Time-to-TKR as a function of number of courses of HA injection. Each individual comparison of an HA User cohort to HA Non-users was significant (p < 0.0001; see Table 3). All HA cohorts had substantially longer time-to-TKR than HA Non-users (all, p < 0.0001), with the weakest significance for patients who got only 1 course of HA injection (χ^2^ = 13,724.6; p < 0.0001).

More courses of HA injection were associated with a longer time-to-TKR in a dose-dependent manner (Fig 2). Patients who received no HA had a mean time-to-TKR of about 0.7 years (median = 0.3 years). With only one course of HA, the mean time to TKR was 1.4 years (χ^2^ = 13,725; p < 0.0001) (median = 1.1 years), while patients who received ≥5 courses delayed TKR by a mean of 3.6 years (χ^2^ = 19,935; p < 0.0001) (median = 3.6 years). Roughly 22.2% of patients with ≥5 HA courses were able to delay TKR by 4.5 years or more. All HA User cohorts were significantly (p < 0.0001) and substantially different from HA Non-users in time-to-TKR.

## Discussion

Among 182,022 patients with knee OA, those who received HA injections showed a significantly longer time-to-TKR (p < 0.0001). More courses of HA treatment were associated with a longer time-to-TKR, in a dose-dependent manner, consistent with our initial hypothesis. Patients who received no HA had a median time-to-TKR of ~0.3 years; with only one course of HA, the median time to TKR was >1.0 year (χ^2^ = 13,725; p < 0.0001); patients who received ≥5 courses delayed TKR by 3.6 years (χ^2^ = 19,935; p < 0.0001).

The strongest evidence suggesting that HA can delay TKR is the clear dose-response relationship between number of HA courses and time-to-TKR (Fig 2). While it is essentially impossible to prove a causal inference linking an event with an outcome [13], the dose-response relationship between HA and delay of TKR (Fig 2) would seem to satisfy many of Hill’s criteria for causation [14]: the relationship between number of HA courses and time-to-TKR is statistically very strong (legend, Fig 2); the smoothness of the curves (Fig 2) suggests intra-patient consistency of treatment response; the selection criteria used (Fig 1) ensure that the relationship between event and outcome has both high specificity and clear temporality; greater use of HA is associated with a longer time-to-TKR (Fig 2); and there is a plausible mechanism linking cause and effect [10].

Our results are consistent with previous research showing that HA can delay TKR. Retrospective analysis of 248 knee OA patients, using TKR as the endpoint during a 6-month follow-up, concluded that HA may have delayed TKR in 80% of patients [15]. Similarly, TKR was avoided or significantly delayed in 15 of 19 patients who were considering surgery prior to HA injection [16]. In a study of patients with Grade IV OA [17, 18], a total of 1,978 courses of HA were given to 1,187 knees, at an average cost of $1,420 per knee, to delay TKR by a median of 2.1 years [17]. Survival analysis of this group showed that TKR was delayed more than 7 years in 75% of HA-treated patients with Grade IV OA [18]. However, only 19% (N = 225) of patients in this study had TKR [17]. The sample size we report is therefore more than 800-fold larger and provides a higher degree of statistical confidence.

There is also recent evidence from the Truven MarketScan database that repeated courses of HA injection can delay TKR in a dose-dependent fashion by a median of 2.6 years, based on a population of 16,589 patients [4]. Claims analysis in the Blue Cross-Blue Shield database for New Jersey showed that mean time-to-TKR after starting HA was approximately 2.5 years, though 75% of patients underwent TKR within 1 year of starting HA [19]. In a Spanish study of 224 patient candidates for TKR, HA use was associated with a delay in need for surgery that averaged 2.7 years [20]. Clinic-based studies also suggest that repeated cycles of HA injection can delay time-to-TKR [21]. There is thus a growing consensus that HA injections can delay TKR by several years [22], and our results are consistent with that conclusion (Table 3; Fig 2).

How can HA injections delay TKR by several years? Synovial fluid in the joint space is a lubricant and shock absorber [23], and early research suggested that injected HA restores the rheological properties of synovial fluid [24]. However, improved shock absorption and lubrication alone do not explain the multi-year improvement associated with HA injection; improvement also depends upon biologic effects on the cartilage and joint space, and perhaps even on pain perception [25]. There is evidence that HA suppresses the production and activity of pro-inflammatory molecules and degradative enzymes, and can alter immune cell function [3, 10, 24]. Histological evidence demonstrates that HA injections can prevent cartilage degradation and may promote cartilage regeneration [10]. Injected HA can also stimulate endogenous HA production and normalize the rheology of synovial fluid in the OA knee [26]. The physiological effects of injected HA are thus associated with a multifactorial mechanism for HA-related symptomatic improvement.

Use of HA for OA patients has become controversial despite widespread use. The Osteoarthritis Research Society International (OARSI) stated in its 2008 guidelines that IA injections of HA “may be useful in patients with knee or hip OA. [HAs] are characterized by delayed onset, but prolonged duration, of symptomatic benefit when compared to IA injections of corticosteroids” [2]. Use of HA for knee OA has been recommended by the American College of Rheumatology (ACR) [27], the European League Against Rheumatism (EULAR) [28], and the Michigan Quality Improvement Consortium (MQIC) [29]. However, recent guidance from OARSI concluded that the benefit of HA was “uncertain” for knee OA, though the effect size for pain relief following HA injection ranged from 0.37 to 0.46 and the overall evidence quality was “good” [30]. Clinical Practice Guidelines from the Veterans Affairs/Department of Defense [31] state that HA “may be considered for patients who have not responded adequately to non-pharmacologic measures and who have an inadequate response, intolerable adverse events, or contraindications to other pharmacologic therapies.” Conversely, a systematic review by the U.S. Bone and Joint Initiative describes HA use as controversial [32], and both the National Collaborating Center for Chronic Conditions (NCC-CC) [33] and the American Academy of Orthopedic Surgeons (AAOS) [8] have recommended against using HA. The AAOS evaluation of evidence in particular has been faulted for misinterpreting the minimum clinically important improvement necessary to recommend HA therapy [34]. Finally, a recent network meta-analysis incorporating 33,243 patients concluded that HA was superior to both NSAIDs (acetaminophen, celecoxib, and naproxen) and placebo (oral and intra-articular) for knee OA pain relief [35].

The formulation of rational treatment algorithms for HA use should take into account whether HA injection alters disease progression [11]. While our results show unequivocally that HA injection is associated with a delay in time-to-TKR by 3.3 years (Fig 2), our results do not address whether there is a direct patient benefit from such delay. One could imagine that at least some patients who postpone TKR may ultimately avoid knee surgery completely, as suggested in a retrospective trial [18].

Might there be a patient benefit from avoiding TKR? Mortality rates following elective TKR have decreased substantially over the last 30 years [36], but TKR is still associated with serious adverse events (AEs). The incidence of several important AEs associated with TKR increased or did not change from 1999 to 2008, including pulmonary embolism, pneumonia, and cardiac complications [37, 38]. AEs affect roughly 6% of Medicare patients receiving TKR and the risk of congestive heart failure and chronic obstructive pulmonary disease roughly double during surgical hospitalization [39]. A recent population-based study used propensity-matching to compare TKR patients with non-TKR controls and concluded the hazard ratio for myocardial infarction was significantly elevated to 8.8 in the immediate post-operative period [40]. The overall surgical infection rate following TKR was 1% in 5,277 surgeries [41], and rates of sepsis and severe sepsis have not declined in recent years [42]; infection rates can be substantially higher in patients who are morbidly obese, diabetic, or younger in age [43]. Finally, up to 1.7% of patients who get TKR need revision surgery because of implant failure [44]. In a large cohort of patients, the unplanned hospital readmission rate following TKR was 4% at 30 days post-surgery and 8% at 90 days [45]. Among Medicare beneficiaries specifically, the 30-day hospital readmission rate was 5% in 2010 [46]. In aggregate, these findings suggest that some patients may not benefit from TKR, though TKR can yield substantial improvements in physical function and general health [47], including conferring a mild cardioprotective effect over at least 2 ensuing years [48]. These conclusions were recently confirmed in a randomized controlled trial that enrolled 95 patients and found that TKR resulted in greater pain relief and functional improvement after 12 months, at the cost of a rate of serious AEs roughly 4-fold higher than the group which received nonsurgical treatment [49].

By comparison, a recent meta-analysis of viscosupplementation for knee OA concluded that local AEs in the injected knee were infrequent; in large RCTs with blinded outcome assessment, 12 of 3,631 patients (0.3%) had a local AE [50]. This study was weakened by the fact that it included many patients from unpublished studies and such data must be considered unverifiable [51]. A more recent meta-analysis that included 4,866 patients, all from peer-reviewed journal papers, concluded that there were no serious AEs related to HA treatment [51]. Thus, HA injection appears to be safe by comparison to TKR [51,52]. Furthermore, a recent meta-analysis [51] concluded that there were large treatment effects 4–26 weeks after HA injection; relative to saline-injected controls, the standardized mean difference following HA injection was an improvement of 0.38–0.43 units for knee pain and of 0.32–0.34 units for knee function (all P < 0.001).

A potential limitation of this study is that patients who did and did not receive HA injection were not identical at baseline (Tables 1 and 4). Though there were statistically significant differences between HA Users and HA Non-users, the sample size in this database was enormous, so differences that were not clinically meaningful could still be statistically significant. For example, CCI scores were calculated by tallying whether a patient had any of 15 possible comorbid illnesses (*e*.*g*., diabetes, myocardial infarction, congestive heart failure, chronic pulmonary disease, peripheral vascular disease, dementia, cerebrovascular disease, etc.), with a maximum possible score of 15 for patients who had all illnesses [53]. HA Users had an average of 0.6 illnesses, while HA Non-users had an average of 0.7 illnesses (Table 1). Data show that 65.5% of HA Users were free of all major disease comorbidities, while 64.4% of HA Non-users were free of all major comorbidities; median number of comorbidities did not differ between HA Users and HA Non-users. A Cox proportional hazards model found no difference in risk of TKR as a function of comorbidity (Table 2). In short, though CCI differences were statistically significant between HA Users and Non-users (p < 0.0001), we do not believe these differences to be clinically meaningful. Another limitation of this study is that patients who were unwilling to undergo surgery were perhaps more likely to look for alternative treatments such as repeat HA injections; thus Fig 2 could simply reflect a reluctance to undergo surgery. However, we do not think this is a serious limitation; if a patient sought to avoid surgery with HA but the HA treatment failed, then the patient would likely go on to surgery anyway [49]. A final weakness of this study is that, if HA injection enabled a patient to avoid TKR entirely, we would not have been sensitive to that outcome. This is because we required TKR to enroll all patients in the database. This could have had the effect of underestimating the efficacy of HA injection. Therefore, we conclude that HA-related delay of TKR is of significant benefit to patients with knee OA.

## Conclusions

We present evidence that HA injection is associated with a significant delay in TKR. Patients who received no HA had a median time-to-TKR of ~0.3 years; with one course of HA, the median time to TKR was >1.0 year (p < 0.0001); patients who received ≥5 courses delayed TKR by 3.6 years (p < 0.0001). The dose-response relationship between number of HA courses and time-to-TKR suggests that there is clinical benefit from HAs. This relationship is statistically strong; there is consistency of treatment response between patients; greater use of HA is associated with a longer time-to-TKR; and there is a plausible mechanism linking cause and effect [10].

## Supporting Information

S1 Text These tables summarize all of the data received from IMS Health. (XLSX)

## References

[1] Cross M, Smith E, Hoy D, Nolte S, Ackerman I, Fransen M, et al The global burden of hip and knee osteoarthritis: estimates from the global burden of disease 2010 study. Ann Rheum Dis. 2014;73(7):1323–30. 10.1136/annrheumdis-2013-204763 24553908

[2] Zhang W, Moskowitz RW, Nuki G, Abramson S, Altman RD, Arden N, et al OARSI recommendations for the management of hip and knee osteoarthritis, Part II: OARSI evidence-based, expert consensus guidelines. Osteoarthritis and cartilage / OARS, Osteoarthritis Research Society. 2008;16(2):137–62. 10.1016/j.joca.2007.12.013 18279766

[3] Cianflocco AJ. Viscosupplementation in patients with osteoarthritis of the knee. Postgrad Med. 2013;125(1):97–105. 10.3810/pgm.2013.01.2618 23391675

[4] Altman R, Fredericson M, Bhattacharyya SK, Bisson B, Abbot T, Yadalam S, Kim M, Lingohr-Smith M. Impact of hyaluronic acid injections on time to total knee replacement. J Knee Surg. 2015;26:(article accepted). 10.1055/s-0035-1568992 26641076

[5] Slover J, Zuckerman JD. Increasing use of total knee replacement and revision surgery. JAMA: the journal of the American Medical Association. 2012;308(12):1266–8. 23011717 10.1001/jama.2012.12644

[6] Losina E, Paltiel AD, Weinstein AM, Yelin E, Hunter DJ, Chen SP, et al Lifetime medical costs of knee osteoarthritis management in the United States: Impact of extending indications for total knee arthroplasty. Arthr Care Res. 2015;67(2):203–15. 10.1002/acr.22412 PMC4422214 25048053

[7] Bruyère O, Cooper C, Pelletier JP, Branco J, Luisa Brandi M, Guillemin F, et al An algorithm recommendation for the management of knee osteoarthritis in Europe and internationally: A report from a task force of the European Society for Clinical and Economic Aspects of Osteoporosis and Osteoarthritis (ESCEO). Semin Arthritis Rheum. 2014;44(3):253–63. 10.1016/j.semarthrit.2014.05.014 24953861

[8] Jevsevar DS. Treatment of Osteoarthritis of the Knee: Evidence-Based Guideline, 2nd Edition. J Am Acad Orthop Surg. 2013;21(9):571–6. 10.5435/JAAOS-21-09-571 23996988

[9] Fraenkel L, Suter L, Weis L, Hawker GA. Variability in recommendations for total knee arthroplasty among rheumatologists and orthopedic surgeons. J Rheumatol. 2014;41(1):47–52. 10.3899/jrheum.130762 24293580 PMC3880398

[10] Moreland LW. Intra-articular hyaluronan (hyaluronic acid) and hylans for the treatment of osteoarthritis: mechanisms of action. Arthritis research & therapy. 2003;5(2):54–67. 12718745 10.1186/ar623 PMC165033

[11] Newberry SJ, Fitzgerald JD, Maglione MA, O'Hanlon C, Booth M, Motala A, et al Systematic Review for Effectiveness of Hyaluronic Acid in the Treatment of Severe Degenerative Joint Disease (DJD) of the Knee. Department of Health and Human Services: RAND Southern California Evidence-Based Practice Center, 2014 26 Nov 14. Report No. 26866204

[12] Health Economics and Outcomes Research. Database Statistical Analysis Plan. Alexandria, VA IMS Health, 2013.

[13] Kleinberg S, Hripcsak G. A review of causal inference for biomedical informatics. J Biomed Inform. 2011;44(6):1102–12. 10.1016/j.jbi.2011.07.001 21782035 PMC3219814

[14] Hill AB. The environment and disease: Association or causation? Proc Roy Soc Med. 1965;58:295–300. 14283879 10.1177/003591576505800503 PMC1898525

[15] Barrett JP, Siviero P. Retrospective Study of Outcomes in Hyalgan-Treated Patients with Osteoarthritis of the Knee. Clinical drug investigation. 2002;22(2):87–97. 10.2165/00044011-200222020-00003 23315396

[16] Neustadt DH. Long-term efficacy and safety of intra-articular sodium hyaluronate (Hyalgan) in patients with osteoarthritis of the knee. Clin Exp Rheumatol. 2003;21(3):307–11. 12846048

[17] Waddell DD, Bricker DC. Total knee replacement delayed with Hylan G-F 20 use in patients with grade IV osteoarthritis. J Manag Care Pharm. 2007;13(2):113–21. 17330972 10.18553/jmcp.2007.13.2.113 PMC10437553

[18] Waddell DD, Joseph B. Delayed Total Knee Replacement with Hylan G-F 20. J Knee Surg. 2014(10 28. [Epub ahead of print]). 10.1055/s-0034-1395281 25349988

[19] Khan T, Nanchanatt G, Farber K, Jan S. Analysis of the effectiveness of hyaluronic acid in prevention of total knee replacement in osteoarthritis patients. AMCP Nexus2014.

[20] Mar J, Romero Jurado M, Arrospide A, Enrique Fidalgo A, Soler López B. [Cost-analysis of viscosupplementation treatment with hyaluronic acid in candidate knee replacement patients with osteoarhritis]. Rev Esp Cir Ortop Traumatol. 2013;57(1):6–14. 10.1016/j.recot.2012.08.006 23594977

[21] Turajane T, Amphansap T, Labpiboonpong V, Maungsiri S. Total knee replacement following repeated cycles of intra-articular sodium hyaluronate (500–730 Kda) in failed conservative treatment of knee osteoarthritis: a 54-month follow-up. J Med Assoc Thai. 2009;92(Suppl 6):S63–S8. 20120667

[22] Abbott T, Altman RD, Dimef R, Fredericson M, Vad V, Vitanzo P, et al Do hyaluronic acid injections delay total knee replacement surgery? Arthritis Rheum. 2013;65:S910–S1.

[23] Fam H, Bryant JT, Kontopoulou M. Rheological properties of synovial fluids. Biorheology. 2007;44(2):59–74. 17538199

[24] Ghosh P, Guidolin D. Potential mechanism of action of intra-articular hyaluronan therapy in osteoarthritis: are the effects molecular weight dependent? Semin Arthritis Rheum. 2002;32(1):10–37. 12219318 10.1053/sarh.2002.33720

[25] Das A, Neher JO, Safranek S. Clinical inquiries. Do hyaluronic acid injections relieve OA knee pain? J Fam Pract. 2009;58(5):281c–e. 19442386

[26] Bagga H D. B, Sambrook P, March L. Longterm effects of intraarticular hyaluronan on synovial fluid in osteoarthritis of the knee. J Rheumatol. 2006;33(5):946–50. 16652425

[27] Hochberg MC, Altman RD, April KT, Benkhalti M, Guyatt G, McGowan J, et al American College of Rheumatology 2012 recommendations for the use of nonpharmacologic and pharmacologic therapies in osteoarthritis of the hand, hip, and knee. Arthritis care & research. 2012;64(4):465–74. 10.1002/acr.21596 22563589

[28] Jordan KM, Arden NK, Doherty M, Bannwarth B, Bijlsma JW, Dieppe P, et al EULAR Recommendations 2003: an evidence based approach to the management of knee osteoarthritis: Report of a Task Force of the Standing Committee for International Clinical Studies Including Therapeutic Trials (ESCISIT). Annals of the rheumatic diseases. 2003;62(12):1145–55. 14644851 10.1136/ard.2003.011742 PMC1754382

[29] MQIC MD. Medical Management of Adults with Osteoarthritis 2013 [cited 2014 4 Dec 14]. Available from: http://mqic.org/pdf/mqic_medical_management_of_adults_with_osteoarthritis_cpg.pdf.

[30] McAlindon TE, Bannuru RR, Sullivan MC, Arden NK, Berenbaum F, Bierma-Zeinstra SM, et al OARSI guidelines for the non-surgical management of knee osteoarthritis. OA Cart. 2014;22(3):363–88. 10.1016/j.joca.2014.01.003 24462672

[31] Brooks DE, Non-Surgical Management of Hip & Knee Osteoarthritis Working Group T. Clinical Practice Guideline for the Non-Surgical Management of Hip & Knee Osteoarthritis In: Veterans Affairs/Department of Defense T, editor. Washington, DC: Office of Quality and Performance & Office of Evidence Based Practice, US Army Medical Command; 2014 p. 1–126.

[32] Nelson AE, Allen KD, Golightly YM, Goode AP, Jordan JM. A systematic review of recommendations and guidelines for the management of osteoarthritis: The Chronic Osteoarthritis Management Initiative of the U.S. Bone and Joint Initiative. Semin Arthritis Rheum. 2014;43(6):701–12. 10.1016/j.semarthrit.2013.11.012 24387819

[33] National Collaborating Centre for Chronic Conditions N-C. Pharmacological management of osteoarthritis Osteoarthritis: National Clinical Guideline for Care and Management in Adults. London: Royal College of Physicians; 2008. 21290638

[34] Bannuru RR, Vaysbrot EE, McIntyre LF. Did the American Academy of Orthopaedic Surgeons osteoarthritis guidelines miss the mark? Arthroscopy: the journal of arthroscopic & related surgery: Official publication of the Arthroscopy Association of North America and the International Arthroscopy Association. 2014;30(1):86–9. 10.1016/j.arthro.2013.10.007 24384274

[35] Bannuru RR, Schmid CH, Kent DM, Vaysbrot EE, Wong JB, McAlindon TE. Comparative effectiveness of pharmacologic interventions for knee osteoarthritis: A systematic review and network meta-analysis. Ann Intern Med. 2015;162(1):46–54. 10.7326/M14-1231 25560713

[36] Lalmohamed A, Vestergaard P, de Boer A, Leufkens HG, van Staa TP, de Vries F. Changes in mortality patterns following total hip or knee arthroplasty over the past two decades: a nationwide cohort study. Arthritis Rheumatol. 2014;66(2):311–8. 10.1002/art.38232 24504803

[37] Kirksey M, Chiu YL, Ma Y, Della Valle AG, Poultsides L, Gerner P, et al Trends in in-hospital major morbidity and mortality after total joint arthroplasty: United States 1998–2008. Anesth Analg. 2012;115(2):321–7. 10.1213/ANE.0b013e31825b6824 22652311

[38] Memtsoudis SG, Mantilla CB, Parvizi J, Stundner O, Mazumdar M. Have bilateral total knee arthroplasties become safer? A population-based trend analysis. Clin Orthop Relat Res. 2013;471(1):17–25. 10.1007/s11999-012-2608-9 23008025 PMC3528907

[39] Huddleston JI, Maloney WJ, Wang Y, Verzier N, Hunt DR, Herndon JH. Adverse events after total knee arthroplasty: a national Medicare study. J Arthroplasty. 2009;24(6 Suppl):95–100. 10.1016/j.arth.2009.05.001 19577884

[40] Lu N, Misra D, Neogi T, Choi HK, Zhang Y. Total joint arthroplasty and the risk of myocardial infarction—A general population, propensity score-matched cohort study. Arthritis Rheumatol. 2015;[Epub ahead of print] 10.1002/art.39246 PMC4581914 26331443

[41] Willis-Owen CA, Konyves A, Martin DK. Factors affecting the incidence of infection in hip and knee replacement: an analysis of 5277 cases. J Bone Joint Surg Br. 2010;92(8):1128–33. 10.1302/0301-620X.92B8.24333 20675759

[42] Rasouli MR, Maltenfort MG, Purtill JJ, Hozack WJ, Parvizi J. Has the rate of in-hospital infections after total joint arthroplasty decreased? Clin Orthop Relat Res. 2013;471(10):3102–11. 10.1007/s11999-013-2949-z 23575808 PMC3773128

[43] Malinzak RA, Ritter MA, Berend ME, Meding JB, Olberding EM, Davis KE. Morbidly obese, diabetic, younger, and unilateral joint arthroplasty patients have elevated total joint arthroplasty infection rates. J Arthroplasty. 2009;24(6 Suppl):84–8. 10.1016/j.arth.2009.05.016 19604665

[44] Meding JB, Ritter MA, Davis KE, Farris A. Meeting increased demand for total knee replacement and follow-up: determining optimal follow-up. Bone Joint J. 2013;95-B(11):1484–9. 10.1302/0301-620X.95B11.32467 24151267

[45] Schairer WW, Vail TP, Bozic KJ. What are the rates and causes of hospital readmission after total knee arthroplasty? Clin Orthop Relat Res. 2014;472(1):181–7. 10.1007/s11999-013-3030-7 23645339 PMC3889434

[46] Cram P, Lu X, Kates SL, Singh JA, Li Y, Wolf BR. Total knee arthroplasty volume, utilization, and outcomes among Medicare beneficiaries, 1991–2010. JAMA: the journal of the American Medical Association. 2012;308(12):1227–36. 23011713 10.1001/2012.jama.11153 PMC4169369

[47] George LK, Hu L, Sloan FA. The effects of total knee arthroplasty on physical functioning and health among the under age 65 population. Value Health. 2014;17(5):605–10. 10.1016/j.jval.2014.04.004 25128054

[48] Ravi B, Croxford R, Austin PC, Lipscombe L, Bierman AS, Harvey PJ, et al The relation between total joint arthroplasty and risk for serious cardiovascular events in patients with moderate-severe osteoarthritis: propensity score matched landmark analysis. Br J Sports Med. 2014;48(21):1580 10.1136/bjsports-2014-f6187rep 25313134

[49] Skou ST, Roos EM, Laursen MB, Rathleff MS, Arendt-Nielsen L, Simonsen O, Rasmussen S. A randomized, controlled trial of total knee replacement. N Engl J Med. 2015;373:1597–606. 10.1056/NEJMoa1505467 26488691

[50] Rutjes AW, Juni P, da Costa BR, Trelle S, Nuesch E, Reichenbach S. Viscosupplementation for Osteoarthritis of the Knee: A Systematic Review and Meta-analysis. Ann Intern Med. 2012;157(3):180–91. 10.7326/0003-4819-157-3-201208070-00473 22868835

[51] Strand V, McIntyre LF, Beach WR, Miller LE, Block JE. Safety and efficacy of US-approved viscosupplements for knee osteoarthritis: a systematic review and meta-analysis of randomized, saline-controlled trials. J Pain Res 2015;8:217–228. 10.2147/JPR.S83076 26005358 PMC4428363

[52] McArthur BA, Dy CJ, Fabricant PD, Valle AG. Long term safety, efficacy, and patient acceptability of hyaluronic acid injection in patients with painful osteoarthritis of the knee. Patient Prefer Adherence. 2012;6:905–10. 10.2147/PPA.S27783 23271899 PMC3526887

[53] Charlson ME, Pompei P, Ales KL, MacKenzie CR. Charlson Comorbidity Index: farmacologiaclinica.info; 2014 [12 December 14]. Available from: http://tools.farmacologiaclinica.info/index.php?sid=37147.

