# Correction: Hyaluronic Acid Injections Are Associated with Delay of Total Knee Replacement Surgery in Patients with Knee Osteoarthritis: Evidence from a Large U.S. Health Claims Database

**DOI:** 10.1371/journal.pone.0148591

**Published:** 2016-01-29

**Authors:** Roy Altman, Sooyeol Lim, R. Grant Steen, Vinod Dasa

There is an error in the Supporting Information where the incorrect database is linked. Please view the correct S1 Text below.

## Supporting Information

S1 Text These tables summarize all of the data received from IMS Health. (XLSX)
